# Multi-center phase II trial of bortezomib and rituximab maintenance combination therapy in patients with mantle cell lymphoma after consolidative autologous stem cell transplantation

**DOI:** 10.1186/s13045-018-0631-3

**Published:** 2018-06-28

**Authors:** Robert W. Chen, Joycelynne M. Palmer, Sarah Tomassetti, Leslie L. Popplewell, Jessica Alluin, Pritsana Chomchan, Auayporn P. Nademanee, Tanya Siddiqi, Ni-Chun Tsai, Lu Chen, Fay Zuo, Rosemarie Abary, Ji-lian Cai, Alex F. Herrera, John J. Rossi, Steven T. Rosen, Stephen J. Forman, Larry W. Kwak, Leona A. Holmberg

**Affiliations:** 1 Department of Hematology/Hematopoietic Cell Transplantation, City of Hope National Medical Center, 1500 E Duarte Road, Duarte, CA 91010 USA; 2 Department of Information Sciences, City of Hope National Medical Center, Duarte, CA USA; 3 Department of Molecular and Cellular Biology, Beckman Research Institute of the City of Hope National Medical Center, Duarte, CA USA; 4 Clinical Trial Office, City of Hope National Medical Center, Duarte, CA USA; 5 Kaiser Permanente Southern California Bone Marrow Transplantation Program, Los Angeles, CA USA; 6 Judy and Bernard Briskin Center for Multiple Myeloma Research, City of Hope National Medical Center, Duarte, CA USA; 7 Toni Stephenson Lymphoma Center, Department of Hematology/Hematopoietic Cell Transplantation, City of Hope National Medical Center, Duarte, CA USA; 8 Clinical Research Division, Fred Hutchinson Cancer Research Center, Department of Medicine, University of Washington, Seattle, WA USA

**Keywords:** Bortezomib, Rituximab, CCND1, MRD, Mantle cell lymphoma, Auto-HCT

## Abstract

**Background:**

Mantle cell lymphoma (MCL) is an aggressive and incurable lymphoma. Standard of care for younger patients with MCL is induction chemotherapy followed by autologous stem cell transplantation (auto-HCT). Rituximab maintenance after auto-HCT has been shown to improve progression-free survival (PFS) and overall survival (OS) in MCL. Bortezomib maintenance therapy has also been shown to be tolerable and feasible in this setting. However, the combination of bortezomib and rituximab as maintenance therapy post-auto-HCT has not been studied.

**Methods:**

We conducted a multicenter, phase II trial of bortezomib given in combination with rituximab as maintenance in MCL patients after consolidative auto-HCT. Enrolled patients (*n* = 23) received bortezomib 1.3 mg/m^2^ subcutaneously weekly for 4 weeks every 3 months (up to 24 months) and rituximab 375 mg/m^2^ intravenously weekly for 4 weeks every 6 months (up to 24 months) for a total duration of 2 years. The primary study endpoint was disease-free survival (DFS).

**Results:**

With a median follow-up of 35.9 months, the 2-year DFS probability was 90.2% (95% CI 66–97), and 2-year OS was 94.7% (95% CI 68–99). The most frequent grade 3/4 toxic events were neutropenia (in 74% of patients) and lymphopenia (in 35%). The incidence of peripheral neuropathy was 48% for grade 1, 9% for grade 2, and 0% for grade 3/4. We also examined the role of quantitative cyclin D1 (CCND1) mRNA in monitoring minimal residual disease.

**Conclusion:**

Combined bortezomib and rituximab as maintenance therapy in MCL patients following auto-HCT is an active and well-tolerated regimen.

**Trial registration:**

ClinicalTrials.gov NCT01267812, registered Dec 29, 2010.

## Background

Mantle cell lymphoma (MCL) is an aggressive lymphoma characterized by chromosomal translocation t(11;14)(q13;q32) resulting in overexpression of cyclin D1. It accounts for approximately 6% of all non-Hodgkin lymphomas and has a median overall survival of less than 10 years [1]. Consolidative autologous stem cell transplantation (auto-HCT) following induction chemotherapy has been shown to prolong progression-free survival (PFS) and is currently the standard of care for younger patients with MCL [2–5]. The benefit of consolidative auto-HCT was demonstrated by the European MCL network in a trial that compared consolidative auto-HCT to maintenance interferon and showed improved PFS in the auto-HCT arm (median of 39 versus 17 months [*P* = .0108]) [2].

Rituximab, an anti-CD20 monoclonal antibody, has been shown to improve PFS as maintenance therapy in patients with MCL not undergoing auto-HCT [6] or undergoing auto-HCT [7–9]. Le Gouill et al. showed in a randomized trial that rituximab maintenance can prolong PFS and overall survival (OS) in patients with MCL post consolidative auto-HCT [7]. Bortezomib therapy has been shown to be tolerable and effective in treating MCL patients in the relapsed/refractory setting [8–14]. Till et al. [10] explored the role of bortezomib as maintenance therapy for patients not undergoing auto-HCT, and Kaplan et al. [15] showed that bortezomib as a maintenance therapy improved PFS when compared to historical controls. However, to date, no study has been conducted to assess the use of combined rituximab and bortezomib maintenance therapy after auto-HCT in MCL patients. We tested the hypothesis that the addition of bortezomib to rituximab post-auto-HCT would be tolerable and demonstrate improved anti-lymphoma activity in an open-label, multicenter phase II trial. In addition, we explored the use of quantitative CCND1 mRNA values to monitor disease.

## Methods

### Patients

This open-label, mutli-center, single-arm study phase II trial (ClinicalTrials.gov identifier NCT01267812) was performed according to the declaration of Helsinki and Good Clinical Practice (GCP) guidelines. The protocol was approved by the ethics committee of each institution, and all patients gave written informed consent. Eligible patients were ≥ 18 years of age with a Karnofsky performance score of ≥ 60% and histologically documented or cytological confirmation of MCL. Presence of cyclin D1 was confirmed by either fluorescence in situ hybridization (FISH) or immunohistochemical staining. Patients had to have undergone auto-HCT, with documented CR between days 60 and 180 post-auto-HCT by either CT or CT/PET, and to have started treatment between days 60 and 180 post-auto-HCT.

Patients with ≥ grade 2 neuropathy prior to receiving bortezomib, prior hypersensitivity reaction to bortezomib, positive serology for HIV or active hepatitis B or C, active cardiac disease, abnormal liver or renal function, or concurrent or previous diagnosis of cancer were not eligible.

### Study design

Patients were given bortezomib 1.3 mg/m^2^ subcutaneously every week for 4 weeks every 3 months (up to 24 months) and rituximab 375 mg/m^2^ intravenously every week for 4 weeks every 6 months (up to 24 months). Both were given for a total of 2 years. CT imaging and/or FDG-PET, as well as bone marrow biopsy, were performed every 6 months for the first 2 years during treatment and then yearly thereafter. Due to slow accrual, the trial was closed prematurely with a total of 23 patients.

### Minimal residual disease monitoring

Quantitation of CCND1 mRNA in samples of peripheral blood collected at baseline and on day 1 of each cycle was used to monitor disease. CCND1 mRNA was assessed using droplet digital PCR technology (ddPCR) on RNA extracted from peripheral blood mononuclear cells (PBMCs) [16, 17]. The MCL cell line JVM2 (ATCC, Manassas, VA), and PBMCs from an untreated patient with MCL involvement served as positive controls. A normal healthy donor served as negative control. Absolute transcript concentrations were normalized to the housekeeping gene, hypoxanthine phosphoribosyltransferase 1 (HPRT1). CCND1 mRNA transcript levels in the untreated MCL patient and the JVM2 cell line were both much higher than the normal healthy donor, but JVM2 was lower than the untreated MCL patient. We chose to set JVM2 as 100% and compared each patient’s CCND1 mRNA level to this.

### Statistical analysis

The primary study endpoint was disease-free survival; secondary trial endpoints included toxicity (NCI CTCAE version 4.0), relapse rate, overall survival, and expression of CCND1 mRNA in minimal residual disease (MRD) monitoring. Based on unpublished data at the time of study design from COH prior to 2010, the historical 2-year disease-free survival (DFS) probability in MCL patients treated with rituximab alone was approximately 60%. This trial was designed to detect a 20% improvement in DFS at 2 years, an improvement that would deem the maintenance regimen sufficiently active. With 34 evaluable subjects, the trial had 80% power, *α* = .05/2-sided, to detect a 20% improvement at 2 years, from 60% (estimated historical; 95% CI not provided) to 80%. Response was assessed according to modified criteria for malignant lymphoma, based on Cheson et al. 2007 criteria [18]. DFS was calculated from the start of treatment to the date of first appearance of disease relapse, or death from any cause. DFS and OS were estimated using the product-limit method of Kaplan and Meier; 95% confidence interval was calculated using Greenwood’s formula. Descriptive statistics were used to summarize CCND1 mRNA data.

## Results

**Fig. 1:**
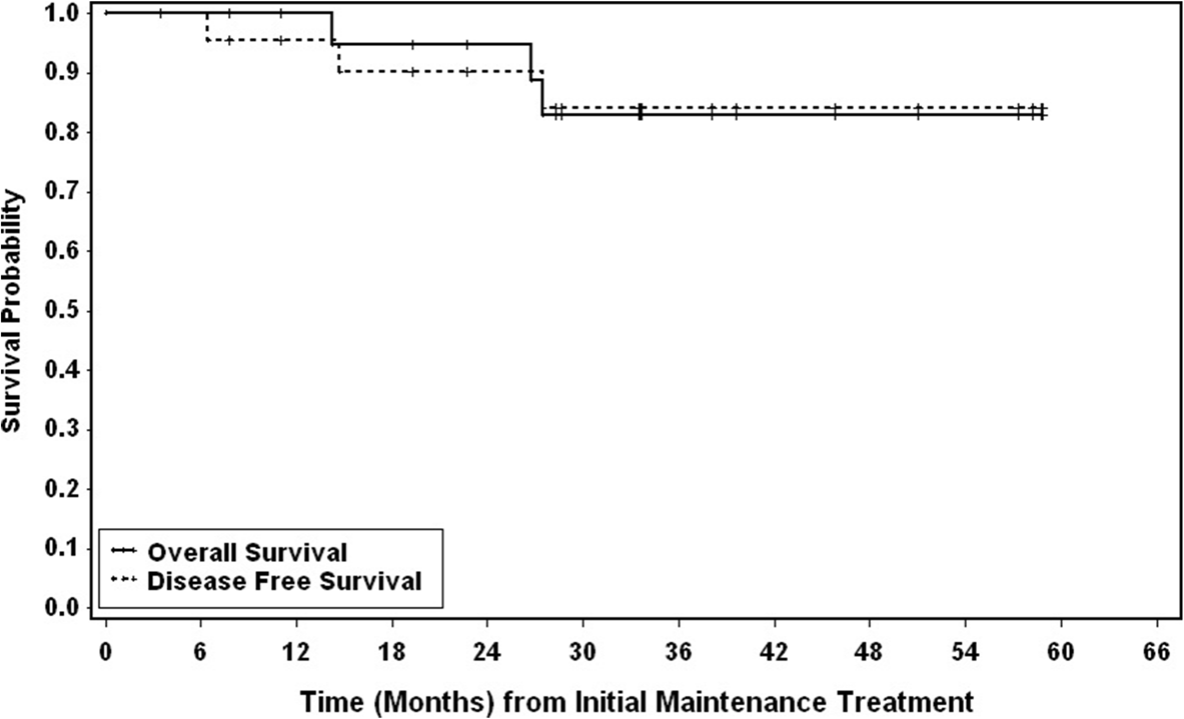
Kaplan-Meier analysis of disease-free and overall survival. The solid line represents overall survival, and the dotted line represents disease-free survival. DFS was calculated from start of treatment to the date of first appearance of disease relapse, or death from any cause. DFS and OS were estimated using the product-limit method of Kaplan and Meier; 95% confidence interval was calculated using Greenwood’s formula

Between September 2011 and October 2016, 23 patients were accrued; all 23 patients were evaluable. The majority of patients were male (96%), received auto-HCT in CR1 (83%), had advanced stage MCL at diagnosis (70% stage IV), and were considered young with a median age of 59 years (range 45–66) (Table 1). Ten out of 23 patients had low-risk disease and 12/23 had intermediate risk disease by the mantle cell international prognostic index (MIPI). The median follow-up was 35.9 months. The 2-year DFS was 93.8% (95% CI 63–99) and the 2-year OS was 92.3% (95% CI 57–99) for the 19 patients who received induction therapy followed by auto-HCT and rituximab/bortezomib maintenance in CR1. The 2-year DFS and OS for all patients were 90.2% (95% CI 66–97) and 94.7% (95% CI 68–99) respectively (Fig. 1).

**Table 1:** Patient characteristics

Characteristics	*n* (%)
Age at initial treatment, years; median (range)	59 (45–66)
Gender	
Female	1 (4)
Male	22 (96)
Disease stage at diagnosis	
I	0 (0)
II	2 (9)
III	4 (17)
IV	16 (70)
Unknown	1 (4)
Extranodal disease at diagnosis	
No	7 (30)
Yes	16 (70)
Time from auto-HCT to initial maintenance treatment (months); median (range)	3.5 (2.3–5.8)
MIPI at diagnosis	
Low	10
Intermediate	12
High	0
Unknown	1
Conditioning regimens for prior auto-HCT	
BEAM	18 (78)
CBV	5 (22)
Induction regimens	
R-bendamustine	3
R-HCVAD/MTX/ARA-C	8
NORDIC	4
RCHOP	3
VR-CAP	2
Ibrutinib	1
Relapsed	
RCHOP followed by R-HCVAD/MTX/ARA-C	1
RCHOP followed by R-bendamustine	1

*Abbreviations*: *BEAM* carmustine, cytarabine, etoposide, and melphalan; *CBV* cyclophosphamide, carmustine, and etoposide; *R*, rituximab; *MTX* methotrexate; *Ara-C* cytarabine; *NORDIC* maximum-strength rituximab, cyclophosphamide, doxorubicin, vincristine, prednisone alternating with rituximab, high-dose cytarabine; *R-HCVAD* rituximab, cyclophosphamide, vincristine, doxorubicin, dexamethasone; *RCHOP* rituximab, cyclophosphamide, doxorubicin vincristine, and prednisone; *VR-CAP* bortezomib, rituximab, cyclophosphamide, doxorubicin, prednisone

The treatment was well tolerated; patients received a median of 8 cycles maintenance bortezomib/rituximab (each cycle = 3 months). The median duration of therapy was 19.9 months. The most common grade 3/4 toxicities in patients included neutropenia (74%), lymphopenia (35%), pneumonia (9%), anemia (9%), skin infections (4%), hypertension (4%), and thrombocytopenia (4%). The incidence of peripheral neuropathy was low with grade 1 events occurring in 11 patients (48%) and grade 2 events occurring in 2 patients (9%) (Table 2). There were no grade 3 or 4 peripheral neuropathies seen. One patient did develop MDS. There were three deaths: two from relapsed MCL and one from allogeneic HCT for MDS.

**Table 2:** Treatment-related adverse events

Category	Toxicity	Number of patients (%)		
		Grade 2	Grade 3	Grade 4
Blood/bone marrow	Leukocyte count decreased	3 (13)	10 (43)	
	Lymphocyte count decreased		7 (30)	1 (4)
	Neutrophil count decreased	1 (4)	7 (30)	10 (43)
	Platelet count decreased	3 (13)	1 (4)	
Cardiac general	Hypertension	5 (22)	1 (4)	
Constitutional symptoms	Chills	1 (4)		
	Fatigue	3 (13)		
	Fever	1 (4)		
Infection	Skin infection	1 (4)	1 (4)	
	Wound infection		1 (4)	
	Lung infection	1 (4)	2 (9)	
Musculoskeletal/soft tissue	Arthralgia	1 (4)		
Neurology	Peripheral sensory neuropathy	2 (9)		
Skin	Rash	1 (4)		

**Fig. 2:**
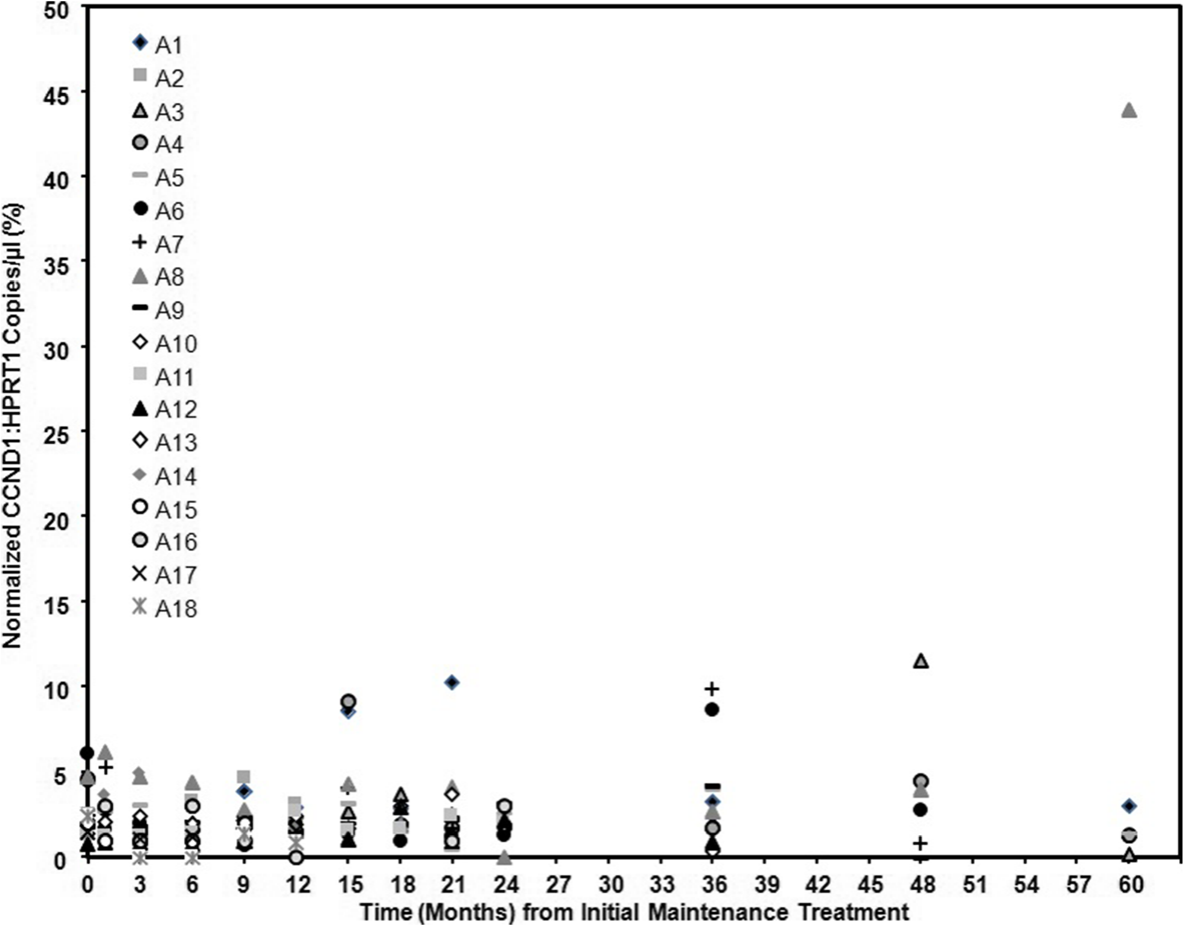
Normalized CCND1 levels in patient peripheral blood samples. CCND1 mRNA was assessed using ddPCR on RNA extracted from PBMCs as described in the “Methods” section. Positive controls were the MCL cell line JVM2, and PBMCs from untreated patient with MCL involvement. Negative control was PBMCs from a healthy donor. Shown are results of single samples from patients A1-A18

Setting JVM2 as positive control at 100%, high CCND1 mRNA (15600%) was detected in an untreated patient with MCL and low CCND1 mRNA (10%) was detected in a normal healthy control. Disease monitoring was performed using CCND1 mRNA at baseline and 12 later time points ranging from 1 month through 60 months on 18 patients treated at City of Hope (Fig. 2). There were a total of 151 samples, with 1–13 samples/time points available per patient and 3–17 samples/patients available per time point. The 151 CCND1 mRNA samples ranged from 0 to 11.6%, with only two samples > 10% (2/151 = 1.3%) and nine samples > 5% (9/151 = 6.0%). Baseline CCND1 mRNA had a mean of 2.7% (range 0.8–6.1%); the average CCND1 mRNA for the 12 later time points had a mean of 2.6% (range 1.8%–4.7%). With 1–13 samples per patient, the average CCND1 mRNA within a patient had a mean of 2.3% (range 0.9–4.7%). Unfortunately, no samples were obtained at or after relapse/progression from the two patients who developed progressive disease, one due to withdrawal of consent prior to relapse and the other due to urgent start of treatment. CCND1 mRNA remained low for all patients in remission.

## Discussion

As MCL is considered an incurable disease with frequent relapses, studies have focused on the use of maintenance therapy to prolong remission either post-induction or post-auto-HCT. While prior studies have shown the importance of maintenance with either rituximab or bortezomib post-auto-HCT [6, 7, 9, 10, 15], this trial is the first to study the combination in this setting. In this multicenter phase II prospective trial, we showed that the combination of rituximab and bortezomib as maintenance therapy post-consolidative auto-HCT is feasible, well tolerated, and yielded a 2-year DFS of 90.2%. Due to the premature closure of the trial, we did not achieve our target accrual of 34 patients. While the trial was ultimately underpowered per the trial design, the estimated DFS of 90.2% (95% CI 66–97) at 2 years exceeded the expected desired result of 80% DFS at 2 years with a lower-bound of the 95% confidence limit that excludes 60%. Therefore, we cautiously conclude that our trial did meet the positive expectation.

As our trial was the first to study the combination regimen of rituximab and bortezomib in a post-auto-HCT population, there was a concern of tolerability. However, we found that the combination was well tolerated; no patients withdrew from the study due to adverse events, and with the exception of hematological toxicities, there were few grade 3 or 4 events. Hematological toxicities were easily managed by the use of intermittent growth factors. Even though the use of bortezomib has been associated with peripheral sensory neuropathy, the incidence of peripheral neuropathy was low in our trial most likely due to dosing schedule and sc route of administration. One patient did develop myelodysplastic syndrome (MDS). However, this was a heavily pretreated patient prior to auto-HCT (RCHOP and R-HyperCVAD), and it took 6 days and two attempts at stem cell mobilization to collect enough stem cells for an auto-HCT. The development of MDS was not attributed to the study combination.

Traditional methods of MRD monitoring include real-time quantitative polymerase chain reaction (PCR) analysis of rearranged immunoglobulin heavy chain (IgH) genes and multicolor flow cytometry [19–21]. These approaches are limited by low sensitivity, failure of marker identification, and requirement for patient-specific strategies. Faham et al. [22] used next-generation sequencing (NGS) which uses locus-specific primer sets of IgH and IgK regions to amplify and sequence cancer-derived clones. These cancer-derived sequences are then used as targets that assess for the presence of MRD in follow-up samples. However, this method still requires baseline patient tumor samples and is relatively cumbersome and expensive to perform. As CCND1 mRNA is expressed in the majority of MCL tumor cells, we explored the use of quantitative CCND1 mRNA from peripheral blood to monitor MRD. As CCND1 mRNA can also be expressed on normal hematopoietic cells, peripheral blood should be more ideal than BM as there would be less contamination [23]. Low CCND1 mRNA was detected in all samples from patients while on maintenance rituximab/bortezomib without radiographic progression of disease. Setting the cutoff at < 10% of control (JVM2 cells), we were able to show 100% specificity of our assay. As this trial started with patients in remission, and there were relatively few relapses, we were not able to correlate progression with rising CCND1 mRNA levels. Therefore, we could not comment on the sensitivity of this method; nevertheless, we have demonstrated its feasibility. We plan to address the issue of sensitivity in a future therapeutic trial where patients will have baseline MCL involvement.

In this study, we demonstrated that bortezomib and rituximab maintenance therapy in MCL patients following consolidative auto-HCT is both tolerable and active. Unfortunately, our study closed prematurely due to slow accrual. Some patients found the four weekly injections (once every 3 months) for a total of 2-year duration laborious, especially as needed to return to the transplant center for the therapy, and chose not to enroll in the trial. However, our study is a proof of concept that addition of proteasome inhibitors to rituximab is feasible and active in the post-auto-HCT setting. As novel oral agents such as Bruton’s tyrosine kinase (BTK) inhibitors, BCL2 inhibitor, and other oral proteasome inhibitors are now available, future studies involving combinations of oral agents with rituximab may be more appealing to patients. Additionally, we demonstrate that low CCND1 mRNA seems to correlate with clinical remission. We plan in future studies to correlate fluctuations in intra-patient CCND1 mRNA as a surrogate marker for disease response in patients with active MCL.

## Conclusions

Our study is limited by the small sample size and single-arm nature but suggests the addition of bortezomib to maintenance rituximab after consolidative auto-HCT may improve DFS and OS in MCL patients.

## Abbreviations

Auto-HCT: Autologous stem cell transplantation
BTK: Bruton’s tyrosine kinase
CCND1: Cyclin D1
ddPCR: Droplet digital PCR technology
DFS: Disease-free survival
FISH: Fluorescence in situ hybridization
GCP: Good Clinical Practice
HPRT1: Hypoxanthine phosphoribosyltransferase 1
IgH: Immunoglobulin heavy chain
MCL: Mantle cell lymphoma
MDS: Myelodysplastic syndrome
MIPI: Mantle cell international prognostic index
MRD: Minimal residual disease
OS: Overall survival
PBMCs: Peripheral blood mononuclear cells
PCR: Polymerase chain reaction
PFS: Progression-free survival
